# Supplementary material for: Does the Heart Fall Asleep?—Diurnal Variations in Heart Rate Variability in Patients with Disorders of Consciousness
Source: Brain Sci. 2022 Mar 11;12(3):375. doi: 10.3390/brainsci12030375 (PMC8946070; doi:10.3390/brainsci12030375)
Supplement: Supplementary file 1 [file brainsci-12-00375-s001.zip › Supplements_Brain Sciences_HRV in patients with DOC_Angerer et al.pdf]

Does the Heart Fall Asleep?—Diurnal Variations in Heart Rate Variability in Patients with Disorders of Consciousness

SUPPLEMENTARY MATERIAL

Monika Angerer [1,2], Frank H. Wilhelm [3], Michael Liedlgruber [3], Gerald Pichler [4], Birgit Angerer [5], Monika Scarpatetti [4], Christine Blume [6,7,†] and Manuel Schabus [1,2,*,†]

1 Laboratory for Sleep, Cognition and Consciousness Research, Department of Psychology, University of Salzburg, 5020 Salzburg, Austria; monika.angerer@plus.ac.at
2 Centre for Cognitive Neuroscience Salzburg (CCNS), University of Salzburg, 5020 Salzburg, Austria
3 Division of Clinical Psychology and Psychopathology, Department of Psychology, University of Salzburg, 5020 Salzburg, Austria; frank.wilhelm@plus.ac.at (F.H.W.); michael.liedlgruber@plus.ac.at (M.L.)
4 Apallic Care Unit, Albert Schweitzer Hospital, Geriatric Health Care Centres of the City of Graz, 8020 Graz, Austria; gerald.pichler@stadt.graz.at (G.P.); monika.scarpatetti@stadt.graz.at (M.S.)
5 Private Practice for General Medicine and Neurology, 8430 Leibnitz, Austria; leibnitz@braindoc.at
6 Centre for Chronobiology, Psychiatric Hospital of the University of Basel, 4002 Basel, Switzerland; christine.blume@unibas.ch
7 Transfaculty Research Platform Molecular and Cognitive Neurosciences, University of Basel, 4055 Basel, Switzerland

* Correspondence: manuel.schabus@plus.ac.at
† These authors contributed equally to this work.

CRS-R Scores

Table S1. CRS-R scores.

Patient ID	Auditory	Visual	Motor	Oromotor	Commuic.	Arousal	Sum Score
P1	0	0	1	0	0	2	3
P2	1	0	1	0	0	1	3
P3	1	0	1	0	0	2	4
P4	4	5	6	3	0	3	21
P5	1	0	1	1	0	1	4
P6	1	0	1	0	0	2	4
P7	0	3	5	0	0	1	9
P8	-	-	-	-	-	-	-
P9	1	1	0	1	0	2	5
P10	1	0	1	1	0	1	4
P11	0	0	0	1	0	1	2
P12	0	0	1	1	0	2	4
P13	3	4	6	1	0	2	16
P14	1	0	2	1	0	2	6
P15	3	5	5	2	1	2	18
P16	4	2	1	1	0	2	10
P17	2	3	2	1	0	2	10
P18	0	3	0	1	0	2	6
P19	0	0	1	1	0	1	3
P20	2	3	2	2	0	2	11
P21	4	5	5	3	1	3	21
P22	0	1	2	1	0	2	6
P23	1	0	1	1	0	2	5
P24	1	1	2	2	0	2	8
P25	0	1	1	1	0	2	5
P26	2	3	2	2	1	3	13

Please note that we could not obtain valid CRS-R assessments in one patient (P8) because not all subscales could be evaluated (i.e., due to eyes being closed and it being impossible to induce eye-opening even when physically stimulating the patient).

Missing Data

Table S2. Amount of missing data from each subject separately for time (i.e., forenoon [8 a.m.–2 p.m.], afternoon [2 p.m.–8 p.m.], night [11 p.m.–5 a.m.]).

	Forenoon		Afternoon		Night	
Patient ID	Frequency	Percentage	Frequency	Percentage	Frequency	Percentage
P1	0	0	0	0	0	0
P2	23	6.39	0	0	0	0
P3	6	1.67	0	0	0	0
P4	7	1.94	0	0	0	0
P5	0	0	0	0	0	0
P6	0	0	21	5.83	0	0
P7	0	0	10	2.78	0	0

P8	0	0	0	0	26	7.22
P9	0	0	0	0	0	0
P10	1	0.28	0	0	0	0
P11	2	0.56	0	0	0	0
P12	0	0	0	0	0	0
P13	0	0	0	0	0	0
P14	0	0	0	0	0	0
P15	0	0	0	0	0	0
P16	0	0	0	0	0	0
P17	0	0	0	0	0	0
P18	0	0	0	0	0	0
P19	0	0	0	0	0	0
P20	0	0	0	0	0	0
P21	0	0	0	0	0	0
P22	0	0	0	0	0	0
P23	0	0	0	0	0	0
P24	0	0	0	0	0	0
P25	0	0	0	0	0	0
P26	0	0	0	0	0	0

Frequency refers to the amount of one-minute segments that are missing. Percentage describes the amount of missing one-minute segments in its relation to the total file length of six hours (i.e., 360 one-minute segments). Missing data is highlighted in grey.

Normality Test Results

Table S3. Shapiro-Wilk tests (*p*-values) for normality for each variable separately for time (i.e., forenoon [8 a.m.–2 p.m.], afternoon [2 p.m.–8 p.m.], night [11 p.m.–5 a.m.]).

Variable	Forenoon	Afternoon	Night
Interbeat interval	0.436	0.551	0.729
Heart rate	0.054	0.061	0.025
RMSSD	0.013	0.003	0.194
Very low frequency	0.012	0.075	0.281
Low frequency	0.005	0.017	0.497
High frequency	0.001	0.008	0.004
Approximate entropy	0.907	0.162	0.193
DfaAlpha	0.067	0.002	0.352
Hurst exponent	0.162	0.056	0.061
Sample entropy 1	0.001	< 0.001	< 0.001
Sample entropy 2	0.212	0.703	0.469
Sample entropy 3	0.054	0.693	0.603
Sample entropy 4	0.092	0.687	0.705
Sample entropy 5	0.079	0.652	0.916
EEG permutation entropy		0.219	0.681
CRS-R sum score	< 0.001		
CRS-R auditory subscale score	< 0.001		
CRS-R visual subscale score	< 0.001		
CRS-R motor subscale score	< 0.001		
CRS-R oromotor subscale score	< 0.001		

CRS-R communication subscale score	< 0.001
CRS-R arousal subscale score	< 0.001
Age	< 0.001
Time since injury	< 0.001

P-values for EEG permutation entropy refer to day (i.e., 8 a.m.–8 p.m.) and night (i.e., 11 p.m.–5 a.m.). *P*-values for CRS-R scores, age and time since injury refer to all time conditions (i.e., forenoon, afternoon, and night). Significant *p*-values (i.e. $p < 0.05$), which indicate that the data is not normal distributed, are marked in grey. Abbreviations: RMSSD = root mean square of successive differences between adjacent heartbeats, DfaAlpha = detrended fluctuation analysis scaling exponent.

Results

Heart Rate

Analyses of the heart rate (HR) of 26 patients revealed a trend towards a main effect for *time* ($F_{WTS}(2)=7.28$, $p=0.053$) and a significant effect for *diagnosis* ($F_{WTS}(1)=5.1$, $p=0.039$), but no significant *time* × *diagnosis* interaction ($F_{WTS}(2)=1.42$, $p=0.515$). Specifically, patients showed a lower HR during night as compared to forenoon ($F_{WTS}(1)=8.26$, $p=0.021$) and afternoon ($F_{WTS}(1)=6.64$, $p=0.024$). No differences could be observed in the patients' HR between fore- and afternoon ($F_{WTS}(1)=0.002$, $p=0.967$; cf. *Figure S1a*). Further, patients with UWS showed a lower HR as compared to patients with (E)MCS ($F_{WTS}(1)=5.1$, $p=0.039$; cf. *Figure S1b*). This effect was also visible when correlating HR and CRS-R sum scores of 25 patients, showing that lower CRS-R sum scores were associated with lower HR during forenoon ($r_\tau(23)=0.34$, $p=0.02$; cf. *Figure S2a*), afternoon ($r_\tau(23)=0.38$, $p=0.009$; cf. *Figure S2b*) and night ($r_\tau(23)=0.28$, $p=0.056$; $b= 1.41$, 95% CI = [0.10, 2.72]; cf. *Figure S2c*).

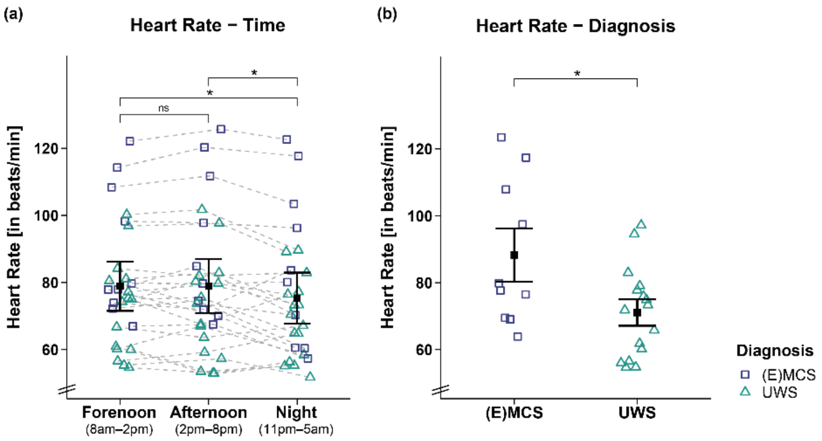

Figure S1. Heart rate (HR) separately for time and diagnosis contrasts. **(a)** While patients' HR was lower during the night as compared to the day (i.e., forenoon, afternoon), it did not differ between fore- and afternoon. **(b)** Patients with UWS showed a lower HR than patients with (E)MCS. Error bars represent the mean and 95% confidence interval. *$p < 0.05$, ns = not significant. Abbreviations: (E)MCS = (emergence from) minimally conscious state, UWS = unresponsive wakefulness syndrome, beats/min = heartbeats per minute.

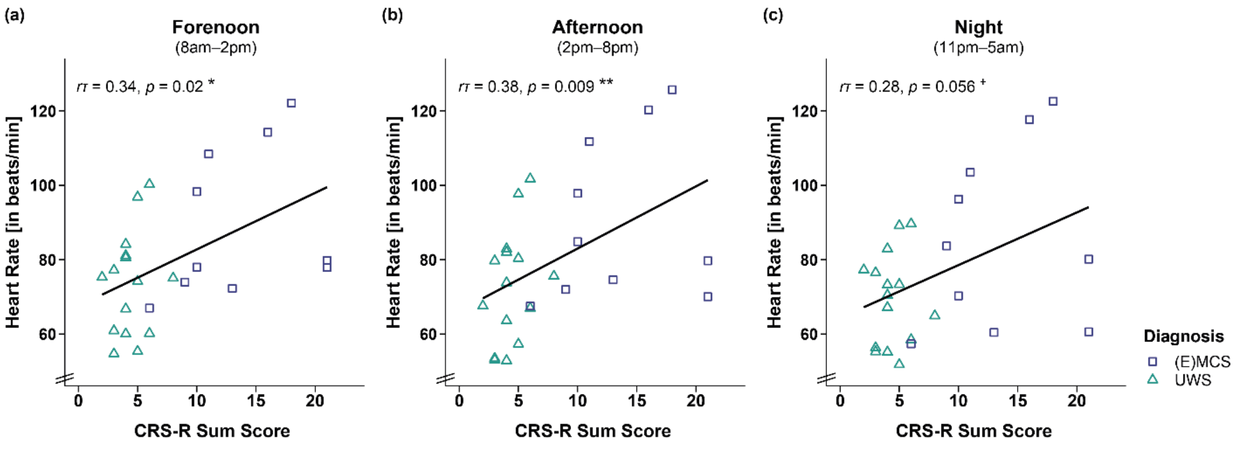

Figure S2. Correlation between heart rate (HR) and CRS-R sum score separately for time. A higher HR was associated with a higher CRS-R sum score throughout the **(a, b)** day (i.e., forenoon, afternoon) and **(c)** night. ** $p < 0.01$, * $p < 0.05$, $^{+}p \leq 0.1$. Abbreviations: (E)MCS = (emergence from) minimally conscious state, UWS = unresponsive wakefulness syndrome, beats/min = heartbeats per minute.

Etiology

Table S4. Wald-type statistic (WTS) of the different HRV parameters separately for the main effects 'time' (i.e., forenoon [8 a.m.–2 p.m.], afternoon [2 p.m.–8 p.m.], night [11 p.m.–5 a.m.]) and 'etiology' (i.e., traumatic vs. non-traumatic brain injury), and the 'time × etiology' interaction (N=26).

Variable	Time	Etiology	Time × Etiology
Interbeat interval	$F_{WTS}(2)=7.37$, $p=0.050$	$F_{WTS}(1)=0.08$, $p=0.778$	$F_{WTS}(2)=5.58$, $p=0.088$
Heart rate	$F_{WTS}(2)=7.82$, $p=0.041$	$F_{WTS}(1)=0.00$, $p=0.949$	$F_{WTS}(2)=3.33$, $p=0.220$
RMSSD	$F_{WTS}(2)=0.50$, $p=0.789$	$F_{WTS}(1)=0.00$, $p=0.961$	$F_{WTS}(2)=1.51$, $p=0.502$
Very low frequency	$F_{WTS}(2)=7.47$, $p=0.046$	$F_{WTS}(1)=0.22$, $p=0.648$	$F_{WTS}(2)=2.19$, $p=0.368$
Low frequency	$F_{WTS}(2)=6.59$, $p=0.065$	$F_{WTS}(1)=0.02$, $p=0.893$	$F_{WTS}(2)=2.90$, $p=0.268$
High frequency	$F_{WTS}(2)=4.08$, $p=0.178$	$F_{WTS}(1)=0.24$, $p=0.619$	$F_{WTS}(2)=2.16$, $p=0.382$
Approximate entropy	$F_{WTS}(2)=20.74$, $p=0.001$	$F_{WTS}(1)=0.30$, $p=0.575$	$F_{WTS}(2)=2.90$, $p=0.277$
DfaAlpha	$F_{WTS}(2)=0.44$, $p=0.816$	$F_{WTS}(1)=4.59$, $p=0.043$	$F_{WTS}(2)=1.29$, $p=0.552$
Hurst exponent	$F_{WTS}(2)=2.36$, $p=0.335$	$F_{WTS}(1)=0.09$, $p=0.772$	$F_{WTS}(2)=2.01$, $p=0.400$
Sample entropy 1	$F_{WTS}(2)=0.41$, $p=0.830$	$F_{WTS}(1)=4.36$, $p=0.049$	$F_{WTS}(2)=1.22$, $p=0.579$
Sample entropy 2	$F_{WTS}(2)=3.79$, $p=0.193$	$F_{WTS}(1)=0.51$, $p=0.477$	$F_{WTS}(2)=2.04$, $p=0.396$
Sample entropy 3	$F_{WTS}(2)=3.4$, $p=0.226$	$F_{WTS}(1)=1.03$, $p=0.316$	$F_{WTS}(2)=1.34$, $p=0.543$
Sample entropy 4	$F_{WTS}(2)=3.26$, $p=0.239$	$F_{WTS}(1)=1.07$, $p=0.304$	$F_{WTS}(2)=0.68$, $p=0.734$
Sample entropy 5	$F_{WTS}(2)=2.43$, $p=0.338$	$F_{WTS}(1)=1.09$, $p=0.300$	$F_{WTS}(2)=0.78$, $p=0.701$

Significant p-values (i.e. $p < .05$) are marked in grey. Abbreviations: RMSSD = root mean square of successive differences between adjacent heartbeats, DfaAlpha = detrended fluctuation analysis scaling exponent.

Sex

Table S5. Wald-type statistic (WTS) of the different HRV parameters separately for the main effects 'time' (i.e., forenoon [8 a.m.–2 p.m.], afternoon [2 p.m.–8 p.m.], night [11 p.m.–5 a.m.]), 'sex' (i.e., male vs. female), and the 'time × sex' interaction (N=26).

Variable	Time	Sex	Time × Sex
Interbeat interval	$F_{WTS}(2)=4.66$, $p=0.156$	$F_{WTS}(1)=2.36$, $p=0.143$	$F_{WTS}(2)=3.89$, $p=0.205$
Heart rate	$F_{WTS}(2)=6.06$, $p=0.099$	$F_{WTS}(1)=1.96$, $p=0.176$	$F_{WTS}(2)=3.82$, $p=0.217$
RMSSD	$F_{WTS}(2)=0.18$, $p=0.920$	$F_{WTS}(1)=0.44$, $p=0.514$	$F_{WTS}(2)=1.41$, $p=0.547$

Very low frequency	$F_{WTS}(2)=12.21$, $p=0.014$	$F_{WTS}(1)=0.09$, $p=0.773$	$F_{WTS}(2)=0.11$, $p=0.950$
Low frequency	$F_{WTS}(2)=12.08$, $p=0.015$	$F_{WTS}(1)=0.53$, $p=0.478$	$F_{WTS}(2)=0.05$, $p=0.977$
High frequency	$F_{WTS}(2)=1.1$, $p=0.622$	$F_{WTS}(1)=1.45$, $p=0.242$	$F_{WTS}(2)=1.82$, $p=0.310$
Approximate entropy	$F_{WTS}(2)=22.29$, $p=0.002$	$F_{WTS}(1)=0.26$, $p=0.607$	$F_{WTS}(2)=2.27$, $p=0.383$
DfaAlpha	$F_{WTS}(2)=0.05$, $p=0.975$	$F_{WTS}(1)=3.92$, $p=0.062$	$F_{WTS}(2)=4.89$, $p=0.135$
Hurst exponent	$F_{WTS}(2)=1.59$, $p=0.495$	$F_{WTS}(1)=0.45$, $p=0.507$	$F_{WTS}(2)=8.47$, $p=0.044$
Sample entropy 1	$F_{WTS}(2)=0.3$, $p=0.883$	$F_{WTS}(1)=1.45$, $p=0.253$	$F_{WTS}(2)=2.78$, $p=0.325$
Sample entropy 2	$F_{WTS}(2)=4.51$, $p=0.159$	$F_{WTS}(1)=0.00$, $p=0.985$	$F_{WTS}(2)=2.17$, $p=0.394$
Sample entropy 3	$F_{WTS}(2)=3.57$, $p=0.229$	$F_{WTS}(1)=0.04$, $p=0.858$	$F_{WTS}(2)=1.89$, $p=0.443$
Sample entropy 4	$F_{WTS}(2)=3.19$, $p=0.268$	$F_{WTS}(1)=0.05$, $p=0.824$	$F_{WTS}(2)=1.41$, $p=0.537$
Sample entropy 5	$F_{WTS}(2)=2.01$, $p=0.428$	$F_{WTS}(1)=0.08$, $p=0.792$	$F_{WTS}(2)=1.39$, $p=0.541$

Abbreviations: RMSSD = root mean square of successive differences between adjacent heartbeats, DfaAlpha = detrended fluctuation analysis scaling exponent.

Entropy Parameters

Table S6. Wald-type statistic (WTS) of the different entropy parameters separately for the main effects 'time' (i.e., forenoon [8 a.m.–2 p.m.], afternoon [2 p.m.–8 p.m.], night [11 p.m.–5 a.m.]) and 'diagnosis' (i.e., E/MCS vs. UWS), and the 'time × diagnosis' interaction (N=26).

**Variable**	**Time**	**Diagnosis**	**Time × Diagnosis**
DfaAlpha	$F_{WTS}(2)=0.48$, $p=0.807$	$F_{WTS}(1)=1.53$, $p=0.228$	$F_{WTS}(2)=0.71$, $p=0.721$
Hurst exponent	$F_{WTS}(2)=3.05$, $p=0.257$	$F_{WTS}(1)=0.22$, $p=0.646$	$F_{WTS}(2)=1.08$, $p=0.604$
Sample entropy 1	$F_{WTS}(2)=0.40$, $p=0.841$	$F_{WTS}(1)=0.44$, $p=0.518$	$F_{WTS}(2)=0.24$, $p=0.901$
Sample entropy 2	$F_{WTS}(2)=4.27$, $p=0.161$	$F_{WTS}(1)=0.07$, $p=0.791$	$F_{WTS}(2)=0.05$, $p=0.980$
Sample entropy 3	$F_{WTS}(2)=3.97$, $p=0.178$	$F_{WTS}(1)=0.01$, $p=0.941$	$F_{WTS}(2)=0.06$, $p=0.972$
Sample entropy 4	$F_{WTS}(2)=3.71$, $p=0.197$	$F_{WTS}(1)=0.12$, $p=0.737$	$F_{WTS}(2)=0.09$, $p=0.958$
Sample entropy 5	$F_{WTS}(2)=2.93$, $p=0.271$	$F_{WTS}(1)=0.21$, $p=0.650$	$F_{WTS}(2)=0.01$, $p=0.996$

Abbreviation: DfaAlpha = detrended fluctuation analysis scaling exponent.

EEG Entropy

Analyses of the EEG permutation entropy (PE) of 14 patients revealed a significant main effect for *time* ($F_{WTS}(2)=9.79$, $p=0.011$) with patients showing higher PE during the day (i.e., 8 a.m.–8 p.m.) as compared to the night (i.e., 11 p.m.–5 a.m.), and a significant *time* × *diagnosis* interaction ($F_{WTS}(2)=5.39$, $p=0.041$), but no significant main effect for *diagnosis* ($F_{WTS}(1)=0.12$, $p=0.733$). Specifically, while patients with (E)MCS showed a lower PE during the night than during the day ($F_{WTS}(1)=21.87$, $p=0.002$), no such day-night difference was observed in patients with UWS ($F_{WTS}(1)=0.25$, $p=0.631$). This has already been shown in Ref. [25]. We ran the analyses again as we only use a subsample of the dataset here.
